# The impact of transcranial direct current stimulation combined with virtual reality-based mindfulness on attention and inhibitory control in healthy individuals

**DOI:** 10.1016/j.ijchp.2025.100631

**Published:** 2025-10-08

**Authors:** Filipa Freire-Santos, Dicle Karacadag, Yasmin Vieira, Mónica Sobral, Vera Mateus, Raquel Guiomar, Perianen Ramasawmy, Andrea Antal, Ana Ganho-Ávila

**Affiliations:** a Faculty of Psychology and Educational Sciences, University of Coimbra, Rua do Colégio Novo, Coimbra 3000-315, Portugal; b Faculty of Psychology, University of Padova, Italy; c Human Developmental Sciences Graduate Program and Mackenzie Center for Research in Childhood and Adolescence, Center for Biological and Health Sciences, Mackenzie Presbyterian University, São Paulo, Brazil; d Center for Research in Neuropsychology and Cognitive Behavioral Intervention, Faculty of Psychology and Educational Sciences, University of Coimbra, Rua do Colégio Novo 3000-115, Coimbra, Portugal; e Non-Invasive Brain Stimulation Lab, Department of Neurology, University Medical Center Göttingen, Georg-August University, Göttingen, Germany

**Keywords:** tDCS, Focused mindfulness, Virtual reality, Sustained attention, Attentional control, Inhibitory control

## Abstract

Combining virtual reality-focused mindfulness (VR-FM) and transcranial direct current stimulation (tDCS) can enhance cognitive performance, offering new insights for scientific research and clinical applications. We aimed to examine the effects of a single session of VR-FM, a single session of tDCS, and their combination on sustained attention, attention control, and inhibitory control.

We conducted a double-blind, controlled, randomized study (*N* = 107) with five groups: VR-FM or VR-mind wandering, paired with active (2 mA for 20 min) or sham tDCS with the anode over F3 and cathode over F4, and a no-intervention control group. Non-specific skin conductance response (nsSCR) was collected during virtual reality, and cognitive performance was measured with Sustained Attention to Response Task (SART) and the Emotional Stroop (EST) after intervention. Differences between groups were not statistically significant in cognitive tasks (all *p*>.05) but we found a main effect of group in nsSCR (*F* (3, 66) = 4.07, *p* = .010, η² = 0.156), with significant differences between VR-FM + tDCS active and VR-MW + tDCS sham (*p* = .014).

Single sessions of VR-FM and tDCS did not significantly impact cognitive performance. However, reduced autonomic activation might be associated with mindfulness. Future studies should include several sessions and consider other individual conditions to understand the factors involved.

## Introduction

Cognitive functions, such as attention and inhibitory control, play a crucial role in one’s daily functioning. As discussed by Posner and Petersen (1990), attention is not a unitary process but a set of interconnected systems - alerting, orienting, and executive control. The alerting system supports sustained attention, which underpins other forms of attention, including divided and selective attention (Slattery et al., 2022). Inhibitory control is the ability to inhibit prevailing emotional and cognitive responses towards goal-directed behaviors (Tiego et al., 2018) and is at the core of optimal psychological functioning (Wessel & Anderson, 2024). Effective emotional and behavioral regulation in daily life often requires redirecting attention away from emotional stimuli and reallocating it to actions that align with one’s goals (Gross, 1998).

Mindfulness is the ability to maintain present-moment awareness (Kabat-Zinn, 2023), noticing internal and external experiences with a non-judgmental attitude (Im et al., 2021). A meta-analysis of randomized controlled trials found that mindfulness interventions produce small but significant effects on attention (*g* = 0.18) and executive control (*g* = 0.18) (Yakobi et al., 2021). Neuroimaging studies further show that mindfulness practice increases blood flow and enhances connectivity in brain regions associated with cognitive control, emotion regulation, and self-awareness, such as the anterior cingulate cortex, insula, and dorsolateral prefrontal cortex (dlPFC; Tang et al., 2015). Moreover, increased connectivity between the dlPFC and the posterior cingulate cortex within the default mode network, has been linked to improved attentional control and reduced mind-wandering (MW) (Sezer et al., 2022).

Focused mindfulness induction is a brief (5–20 mins), guided practice (Gill et al., 2020) where individuals are instructed to concentrate their attention on a particular object or experience, actively avoiding MW (Lutz et al., 2008). However, evidence on the effects of a single mindfulness induction on cognition is mixed. Some reviews report benefits for cognition and emotional regulation (Howarth et al., 2019; Leyland et al., 2019), while others found no significant cognitive improvements (Gill et al., 2020). These heterogeneous results may be due to different brain processes involved in mindful meditation between mindfulness experts and beginners. In fact, while experts use bottom-up processes during mindful meditation, beginners seem to engage mindfulness primarily through top–down processes involving increased prefrontal activity (Chiesa et al., 2013). This difference raises the question of whether a single session is sufficient to produce measurable benefits.

Transcranial direct current stimulation (tDCS) has shown promise in improving cognitive functions (Wolkenstein et al., 2013). For example, a single session of anodal stimulation over the left dorsolateral prefrontal cortex (dlPFC) can modulate attention (Miler et al., 2017; Silva et al., 2017) and inhibitory control (Angius et al., 2019) in both clinical and non-clinical populations. Although, other findings challenge these outcomes (Horvath et al., 2015) a recent systematic review by Divarco et al. (2023), supports that the combination of mindfulness and tDCS in one single session has potential to enhance cognitive functioning.

Virtual reality (VR)-based mindfulness training may be more effective than conventional mindfulness training in improving cognition and emotion regulation (Ma et al., 2023), with gamification elements in VR enhancing participants’ attention and motivation through a more immersive and engaging experience (Arpaia et al., 2022). For example, Mitsea et al. (2022) found that mindful breathing combined with VR enhanced sustained attention and attentional control by facilitating the involuntary allocation of attentional resources. Similarly, Argüero-Fonseca et al. (2022) reported improvements in attention, memory, and motivation through the use of VR and gamification. In this study, we aimed to investigate the effects of VR-FM combined with anodal tDCS over the left dlPFC (F3-F4 montage; 2 mA for 20 min) on sustained attention, attentional control, and inhibitory control in a non-clinical adult sample.

To investigate our hypotheses, we collected self-report measures of subjective experience and psychological state, non-specific skin conductance response (nsSCR), and cognitive performance. We expect that this combination will lead to (1) faster reaction times (RT) indicating behavioural changes in sustained attention performance; (2) faster RT for emotional stimuli, reflecting improvements in cognitive control and reduced interference from unwanted distractors; and that (3) behavioural changes in attentional control will be accompanied by psychophysiological correlates, indexed nsSCR.

## Materials and methods

This randomised, double-blinded, and sham-controlled study was approved by the local Ethical Committee ([blinded]: 91/R_2) and performed in accordance with the Declaration of Helsinki and its revisions. Participants were informed about the research objectives and procedures, the voluntary nature of their participation, the risks, and their right to withdraw at any time.

### Participants

Healthy mindfulness novices (as defined by Wang et al., 2023), university students aged 18–50, fluent in Portuguese, and with at least an intermediate level of proficiency in English were recruited. Participants were excluded if they had (a) current or previous history of psychiatric or neurological diagnosis; (b) ongoing psychopharmacological medication; (c) history of epilepsy; (d) metal items/fragments (except titanium) or electronic implants (e.g. pacemaker) in the brain/skull, neck, and chest; (e) history of head surgery, current or previous head injuries, and/or resulting disorders of consciousness; (f) dermatological conditions on the scalp; and (g) uncorrected vision or color-blindness. Participants were requested to refrain from smoking and consuming alcohol and caffeinated and to abstain from engaging in mindfulness practices or sports within two hours prior to participation.

A sample size of 90 participants was estimated from a power analysis using G*Power 3.1 (Faul et al., 2007), based on analysis of covariance and considering five intervention groups, effect size of 0.4 (Gao & Zhang, 2023), probability error of 0.05, power of 0.85 and 6 covariates. To anticipate loss of participants and/or data, we further accounted for 20 % of the attrition rate and recruited 17 additional participants to the study (*N* = 107).

### Study design and procedures

**Fig. 1:**
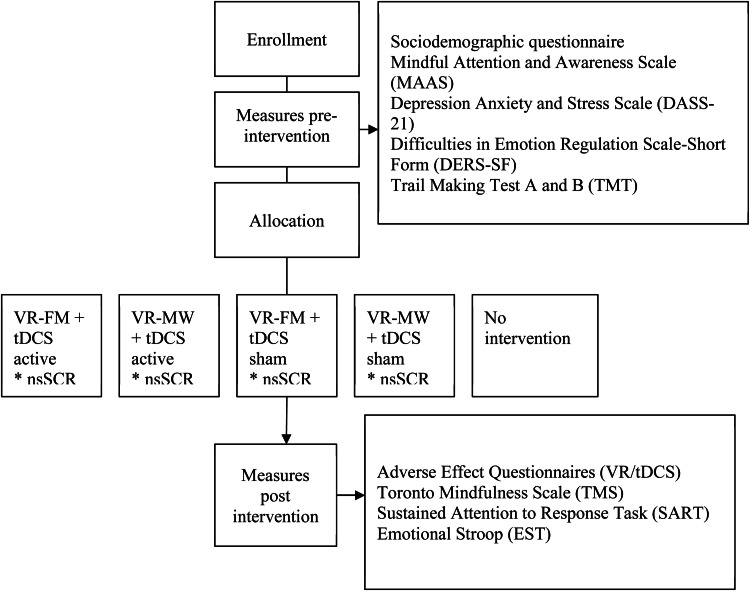
*Study design*. *Note.* tDCS = transcranial direct current stimulation; VR-FM = virtual reality focused mindfulness; VR-MW = virtual reality mind wandering; nsSCR = non-specific skin conductance response.

Participants completed the inclusion/exclusion questionnaire online (LimeSurvey® GmbH, n.d). Eligible individuals were scheduled for an in-person session to receive study details, sign the consent form, and complete sociodemographic, psychological, and pre-tDCS adverse effects questionnaires as well as a baseline cognitive performance assessment. Participants underwent a 2-minute familiarisation session with the virtual environment followed by the experimental protocol - a 20-minute combined intervention during which nsSCR was recorded. Participants were randomly allocated to one of the five groups: VR-FM + tDCS active; *n* = 21 (anodal tDCS over the left dlPFC at 2 mA applied during VR-FM), VR-MW + tDCS active; *n* = 22 (anodal dlPFC-tDCS at 2 mA applied during VR-MW), VR-FM + tDCS sham; *n* = 21 (sham tDCS applied during VR-FM); VR-MW + tDCS sham; *n* = 22 (sham tDCS applied during VR-MW), and no-intervention (*n* = 21). The study was double-blinded (participants and investigators) with regards to tDCS (sham vs active) and single-blinded (participants only) regarding the VR task (VR-FM vs VR-MW). An external researcher conducted the block randomisation with stratification by sex, using Research Randomizer (www.randomizer.org). Participants completed post-tDCS and post-VR adverse events questionnaires, a state mindfulness questionnaire, and a cognitive performance assessment (Fig. 1).

### VR mindfulness and mind-wandering environments

TRIPP (TRIPP Inc., Los Angeles, USA, www.tripp.com) was used to induce VR-FM or VR-MW through Oculus Rift S VR System (Meta Platforms, Inc., CA, USA) which features a 5.5″ fast-switching LCD with a 2560 × 1440 resolution (1280 × 1440 per eye; Schrempf et al., 2022). For the VR-FM condition, we used the "Focus" environment, designed to engage participants in the present moment through attention-based gameplay in a naturalistic scenario and added guided audio on "Staying Focused" (20 min). Participants’ attention was engaged with the request to move their heads to collect golden pieces. In the VR-MW condition (control), the "Calm" environment provided a 20 min naturalistic setting with no audio guidance. Participants were instructed to let their mind wander.

### Transcranial direct current stimulation

A constant direct current of 2 mA was applied for 20 min via a Sooma tDCS™ battery-driven stimulator (Sooma Medical, 2014) with a proprietary Sooma headgear. Two 25 cm^2^ round silicon electrodes mixed with Ag/Al maintained contact with the scalp via 0.9 % isotonic saline-soaked hydrogel pads. Each cap was fitted for anodal tDCS over the left dlPFC (anode at F3, cathode at F4; Herwig et al., 2003), as this montage is the most frequently used in studies combining mindfulness and tDCS (Divarco et al., 2023), showing potential to modulate mindfulness related processes that underlie cognitive performance (Pimenta et al., 2021; Sefat et al., 2022). For the active tDCS, a constant current of 2 mA was delivered for 19 min and 23 s, with 17 s ramp-up and 20 s ramp-down. Sham stimulation consisted of 17 s ramp-up from 0 mA to 2 mA and 17 s ramp-down to 0.3 mA. 0.3 mA constant current was delivered for 19 min 23 s, and 3 s ramp-down at the end of the session, which was previously validated (Hyvärinen et al., 2016; Ramasawmy et al., 2024). tDCS was concurrently applied while participants experienced one of the VR environments (Section 2.2). At the end of the session, we asked participants to guess whether they received active or sham stimulation to assess the effectiveness of the blinding procedure.

### Measures

#### Primary outcomes. *neuropsychological tasks*

The Emotional Stroop Task (EST; Bermonti, 2019) was used to assess attentional control to emotional and non-emotional information. Participants named the colours (red, green, blue) of 144 valence-related words (positive, negative, neutral) from the European Portuguese ANEW (Soares et al., 2012). Participants responded to the stimuli using labelled keys on the keyboard. The EST included 24 practice trials and 16 blocks of 9 trials. Participants were allowed to rest after each 36 trials. The Sustained Attention to Response task (SART; Stothart, 2015). During SART, participants were presented with a digit (ranging from 1 to 9) at the center of a black screen, followed by a mask. Participants were instructed to press the space key immediately upon seeing each digit on the screen (target; go trials), except when the digit 3 appeared (non-target; no-go trials). The practice stage consisted of 18 trials, providing feedback on accuracy. Participants completed 6 blocks of 45 trials (5 font sizes × 9 digits; randomised presentation order), without feedback. The font size of the digits varied randomly between trials (1.20, 1.80, 2.35, 2.50, or 3.00 cm), with each size used 72 times/block. The tasks were presented in Psychopy (Peirce et al., 2022; version 2023.2.3).

#### Secondary outcomes. *psychophysiological data*

Non-specific SCR was measured using the Shimmer3 GSR+ Unit (Shimmer Research Ltd., Dublin, Ireland). Sensors were placed on the index and middle fingers of the non-dominant hand, at a 128 Hz sampling rate. Skin conductance signals were downsampled to 32 Hz and smoothed using a Gaussian window of 200 ms (Benedek & Kaerbach, 2010). Continuous phasic activity was estimated via Continuous Decomposition Analysis (Benedek & Kaerbach, 2010), with tau values optimised to minimise errors. From the CDA-derived SCR list, peak-to-trough amplitudes of individual phasic responses were obtained and averaged within early, middle, and late phases of the VR session. These average amplitudes were used for statistical comparisons.

#### Post-experiment state mindfulness

The Toronto Mindfulness Scale (TMS; Karacadag et al., 2025) comprises 13 items rated on a 5-point Likert scale. Higher scores indicate greater overall state mindfulness.

#### Adverse events

Used to assess the occurrence and severity of side or adverse events on a 10-point rating scale (1: absent, 10: severe; Thair et al., 2017).

#### Baseline cognitive performance

The Trail Making Test A and B (TMT; Cavaco et al., 2013) were used to measure cognitive performance at baseline. Faster completion times indicate better performance. The test has shown good reliability for assessing selective attention and cognitive flexibility (Cavaco et al., 2013).

#### Baseline self-report questionnaires

We collected sociodemographic data and mindfulness experience. Trait mindfulness was assessed using the 15-item Mindfulness Attention Awareness Scale (MAAS) short version (Gregório & Pinto-Gouveia, 2013), answered on a 6-point Likert scale. Higher scores indicate greater mindfulness trait. Depression, anxiety, and stress symptoms were measured using the 21-item Depression, Anxiety and Stress Scale (DASS-21; Pais-Ribeiro et al., 2004). Higher scores indicated higher levels of psychological symptoms. Emotion regulation was evaluated using the Difficulties in Emotion Regulation Scale-Short Form (DERS-SF; Moreira et al., 2020), with 18 items covering six subscales: Non Acceptance of Emotion Responses; Difficulties in Engaging in Goal Behaviors; Impulse Control Difficulties; Lack of Emotion Awareness; Limited Access to Emotion Regulation Strategies; and Lack of Emotion Clarity. Higher scores indicate greater difficulties in emotion regulation.

### Statistical analyses

Statistical analyses were conducted using IBM SPSS Statistics (IBM Corp., 2023, Version 29.2 for Windows, Armonk, NY, USA); R (R Core Team, 2023, Version 4.3.0, Vienna, Austria), RStudio (RStudio Team, 2024, Version 2024.04.1 + 748, Boston, MA, USA), with *lmerTest, lme4, psych, car* and *emmeans* packages; MATLAB R2023b (MathWorks, 2023), and Ledalab 3.4.9 (Ledalab, 2023, Version 3.4.9, http://www.ledalab.de). The significance level was set at *p* < .05.

To control for baseline differences, one-way ANOVAs were conducted for continuous variables and Chi-square tests for categorical variables. Intervention effects on state mindfulness were assessed with one-way ANOVA on TMS scores.

To measure the effects of the combined intervention on nsSCR, we conducted a two-way mixed model ANOVA with intervention (VR-FM + tDCS active; VR-MW + tDCS active; VR-FM + tDCS sham; VR-MW + tDCS sham) as the independent factor and time (early, middle, and late) as the repeated factor. Sixteen participants were excluded from nsSCR analysis due to some data failing to be recorded (*n* = 5) and inability to downsample data collected at non-standard sampling rates (*n* = 11), reducing the statistical power of the nsSCR analyses. Bonferroni corrections were applied for pairwise comparisons.

Generalised Linear Mixed-Effects Models (GLMM) were employed to examine reaction times (RT) across different word valences in EST, with covariate adjustments and a forward approach for covariate entry (Ganho-Ávila et al., 2023): Model 1-unconstrained, without covariates; Model 2-included sociodemographic covariates of age and gender; Model 3-full model with the covariates that better contributed to the model (age, gender, trait mindfulness [MAAS], difficulties in emotional processing [DERS-SF; Lack of Emotion Awareness subscale]; attentional baseline performance [TMT-A] and anxiety symptoms [DASS-21; Anxiety subscale]. Model fit was compared using the Akaike Information Criterion (AIC) (Sakamoto et al., 1986) and marginal *R*² was used to estimate the explained variance by the fixed factors (Nakagawa & Schielzeth, 2013). Variance inflation factor (VIF) values were used to assess multicollinearity (Shrestha, 2020). VIF values between 1 and 5 suggest moderate correlation between the variables, while values over 10 indicate high multicollinearity (Belsley, 1991).

For group differences in SART performance, we analysed mean RT to correct responses, errors of commission, and intra-individual RT variability (ICV). For RT, only correct responses to non-target stimuli were included, excluding RTs under 110 ms and over 1000 ms (Berger & Kiefer, 2021; Grosjean et al., 2001). Commission error rate, reflecting failures in inhibitory control, was calculated as errors on no-go trials divided by total no-go trials (Isbel et al., 2020). ICV, indicating fluctuations in attention, was calculated by dividing the standard deviation of RTs by the mean RT for non-target trials (Isbel et al., 2020). We conducted Chi-square tests to estimate adverse effects group differences.

## Results

### Baseline measures and blinding

One hundred and seven participants (81 women) completed the experimental procedure (Table 1). No significant differences were found between groups across baseline sociodemographic, cognitive, and psychological functioning variables. The results showed that participants could not reliably identify to which groups they were allocated, χ² (2, 86) = 8.10, *p* = .231, with 27 % correct guesses.

**Table 1:** Clinical and sociodemographic characteristics of the participants across groups at baseline.

	VR-FM + tDCS active (*n* = 21)	VR-MW + tDCS active (*n* = 22)	VR-FM + tDCS sham (*n* = 21)	VR-MW + tDCS sham (*n* = 22)	No intervention (*n* = 21)	*F*^a^/χ²	*p*-value
Gender (n, %, Women)	16 (76 %)	16 (73 %)	17 (81 %)	16 (73 %)	16 (76 %)	0.53	.970
Mindfulness practice						5.01	.757
Never or rarely	15 (71 %)	15 (68 %)	17 (81 %)	20 (91 %)	16 (76 %)		
Occasionally	5 (24 %)	6 (27 %)	4 (19 %)	2 (9 %)	4 (19 %)		
Regularly	1 (5 %)	1 (5 %)	0 (0 %)	0 (0 %)	1 (5 %)		
Age (years)	22.33 (7.90)	21.00 (5.27)	20.71 (5.41)	20.36 (1.97)	20.33 (1.85)	0.57	.688
DERS-SF							
Awareness	5.38 (1.47)	5.91 (2.27)	6.67 (1.85)	6.18 (2.26)	5.81 (1.81)	1.24	.299
MAAS	4.11 (0.65)	3.99 (0.59)	3.69 (0.90)	3.91 (0.68)	3.71 (0.76)	1.32	.266
DASS-21							
Depression	5.81 (4.64)	6.00 (5.16)	9.05 (7.00)	7.82 (8.02)	8.00 (8.08)	0.90	.465
Anxiety	4.48 (4.00)	5.82 (5.98)	6.95 (4.88)	5.64 (5.00)	7.24 (7.58)	0.82	.515
Stress	9.81 (5.55)	11.91 (6.55)	11.81 (6.54)	12.55 (7.15)	14.29 (7.19)	1.24	.300
TMT-A time (s)	17.33 (4.08)	19.41 (4.48)	16.57 (5.13)	17.91 (4.72)	19.38 (5.85)	1.41	.237
TMT-A errors	0.10 (0.30)	0.09 (0.29)	0.05 (0.22)	0.09 (0.29)	0.05 (0.22)	0.18	.949
TMT-B time (s)	35.38 (10.7)	50.00 (19.4)	44.24 (21.7)	43.86 (14.6)	44.33 (17.9)	1.96	.107
TMT-B errors	0.33 (0.58)	0.32 (0.48)	0.57 (0.87)	0.23 (0.43)	0.33 (0.73)	0.86	.489

*Note.* Except for gender and mindfulness practice, values are listed as mean (*SD*)

a *F*(4, 102); χ2 = Chi-square test; tDCS = transcranial direct current stimulation; VR-FM = virtual reality focused mindfulness; VR-MW = virtual reality mind wandering; DERS-SF = Difficulties in Emotion Regulation Scale; Awareness = Lack of Emotion Awareness subscale (DERS-SF); MAAS = Mindful Attention and Awareness Scale; DASS-21 = Depression Anxiety and Stress Scale; Depression = Depression subscale (DASS-21); Anxiety = Anxiety subscale (DASS-21); Stress = Stress subscale (DASS-21); TMT-*A* = Trail Making Test Part A; TMT-*B* = Trail Making Test Part B.

### Primary outcomes

Model 3 best fitted the data (AIC = 2554.40), explaining 23 % of the variance in EST performance. RTs across groups were not statistically different compared to the reference group (VR-FM + tDCS active). Only the VR-MW + tDCS active group approached significance (*p* = .080), showing a tendency for longer RTs. Words’ valence did not affect RTs, and none of the interaction terms were significant (Table 2).

**Table 2:** Generalised Linear Mixed-Effects Models - Predictors of reaction time, controlling for covariates.

	Estimated coefficient	*SE*	*t* value	Pr(>|*t*|)
(intercept)	318.58	84.94	3.75	< 0.001⁎⁎⁎
Groups (Ref. FM + tDCS active)				
VR-MW + tDCS active	45.16	25.46	1.77	.080
VR-FM + tDCS sham	−17.53	26.72	−0.66	.514
VR-MW + tDCS sham	13.20	25.70	0.51	.609
Valence (Ref. Negative-valence words)				
Neutral-valence words	3.38	6.63	0.51	.611
Positive-valence words	0.66	6.63	0.10	.921
Random-effects SD	0.73			
AIC				
Model 1 (no covariates)	2587.52			
Model 2 (with sociodemographic variables)	2574.38			
Model 3 (with all covariates of interest)	2554.40			
R^2^				
Model 1 (no covariates)	0.06			
Model 2 (with sociodemographic variables)	0.13			
Model 3 (with all covariates of interest)	0.23			

*Note*. Ref. = Category of reference; SE = Standard error; Estimates, SE, t values and p value of the full model. tDCS = transcranial direct current stimulation; VR-FM = virtual reality focused mindfulness; VR-MW = virtual reality mind wandering.

⁎⁎⁎ *p* < .005.

Results indicated no significant differences in SART performance across groups for any of the outcome measures (for RT, *F*(4102) = 0.22, *p* = .93; for Error of Commission, *F*(4102) = 0.42, *p* = .79; and for ICV, *F*(4102) = 0.32, *p* = .86); Table 3)

**Table 3:** Characterization and differences of the SART Performance.

	FM + tDCS active (*n* = 21)	MW + tDCS active (*n* = 22)	FM + tDCS sham (*n* = 21)	MW + tDCS sham (*n* = 22)	No İntervention (*n* = 21)	*F*^a^	*p*-value
RT (ms)	406.43 (84.6)	420.88 (90.54)	403.96 (68.93)	404.25 (75.05)	397.7 (105.51)	0.22	.93
Commission Error	0.33 (0.19)	0.34 (0.18)	0.38 (0.19)	0.39 (0.19)	0.39 (0.25)	0.42	.79
ICV (ms)	265.12 (72.09)	262.53 (73.91)	279.38 (69.34)	276.25 (66.36)	259.13 (79.91)	0.32	.86

*Note.* Values are listed as mean (SD).

a *F*(4, 102); *RT =* mean of reaction time to correct answers; ICV = intra-individual reaction time variability; *n =* number of participants in each condition*; M =* mean*; SD =* standard deviation; Min = minimum; Max = maximum; *F* = *F*-statistics; *p* = statistical significance.

### Secondary outcomes

Repeated measures ANOVA showed no main effect of session moment (early, middle, late; F(3, 66) = 2.41, *p* = .098, η² = 0.07). The main effect of group was significant (F(3, 66) = 4.07, *p* = .010, η² = 0.156). No significant group x moments interaction was found (F(3, 66) = 1.03, *p* = .408, η² = 0.046). Pairwise comparisons revealed a significant difference in nsSCR between VR-FM + tDCS active and VR-MW + tDCS sham groups (*p* = .014). No other pairwise comparisons were statistically significant. Mean nsSCR values were −0.09 (SD = 0.19) for the VR-FM + tDCS active group, 0.01 (SD = 0.11) for the VR-MW + tDCS active group, −0.07 (SD = 0.09) for the VR-FM + tDCS sham group, and 0.06 (SD = 0.17) for the VR-MW + tDCS sham group.

### TMS outcomes

The one-way ANOVA on the total TMS scores post-intervention revealed no differences between groups, *F*(3, 82) = 0.18, *p* = .912, η^2^ =0.03.

### Adverse events

We found no significant group differences in adverse events (Table 4).

**Table 4:** Post-intervention adverse effects.

	VR-FM + tDCS active (*n* = 21)	VR-MW + tDCS active (*n* = 22)	VR-FM + tDCS sham (*n* = 21)	VR-MW + tDCS sham (*n* = 22)	*p*-value
Headache	2 (10 %)	0 (0 %)	1 (5 %)	2 (9 %)	.562
Neck pain	2 (10 %)	4 (18 %)	3 (14 %)	1 (5 %)	.571
Back pain	3 (14 %)	3 (14 %)	2 (10 %)	1 (5 %)	.735
Blurred vision	1 (5 %)	1 (5 %)	1 (5 %)	1 (5 %)	1.00
Scalp irritation	4 (19 %)	3 (14 %)	5 (24 %)	3 (14 %)	.777
Tingling sensation	2 (10 %)	5 (23 %)	1 (5 %)	0 (0 %)	.054
Itching	6 (29 %)	6 (27 %)	4 (19 %)	3 (14 %)	.629
Accelerated heartbeat	1 (5 %)	1 (5 %)	0 (0 %)	0 (0 %)	.868
Burning sensation	1 (5 %)	3 (14 %)	1 (5 %)	1 (5 %)	.714
Dizziness	1 (5 %)	2 (9 %)	1 (5 %)	1 (5 %)	1.00
Fatigue	2 (10 %)	0 (0 %)	2 (10 %)	5 (23 %)	.106

*Note.* Values are reported as n ( %), indicating how often each adverse effect was reported. Categorical variables were analysed using the Chi-square test. tDCS = transcranial direct current stimulation; VR-FM = virtual reality focused mindfulness; VR-MW = virtual reality mind wandering.

## Discussion

Our findings contrast with previous literature that indicates that a single session of mindfulness can lead to cognitive improvements. Larson et al. (2013) observed enhancements in cognitive control using the Flanker task following a 14-minute Mindfulness of Breathing exercise; and Jaiswal et al. (2020), reported improvements in inhibitory control after a 20-minute session of breath-focused attentional training. Similarly, Sleimen-Malkoun et al. (2023) demonstrated that a brief 10-min audio meditation can positively impact cognitive performance, regardless of participants' prior meditation experience. The lack of significant effects of our intervention may be attributed to the brief nature of the mindfulness meditation session as it is well-established that executive functions can be influenced by the duration and intensity of mindfulness interventions (Ahne & Rosselli, 2024). On the other hand, our findings suggest that different modalities of mindfulness meditation may have varying impacts on cognitive outcomes, warranting further investigation.

The lack of group differences aligns with the literature indicating inconsistent cognitive effects of single tDCS sessions in healthy populations. For instance, Yu et al. (2024) noted that while tDCS applied to the middle temporal cortex enhances decision-making and visuomotor skills in athletes, targeting the dlPFC does not yield cognitive improvements. Kaminski et al. (2024) reported no benefits in sequential skill learning with single tDCS sessions on the dlPFC and concluded that single tDCS session protocols do not improve working memory.

Moreover, the efficacy of anodal tDCS over the left prefrontal regions may vary considerably depending on the participant's level of arousal, potentially accounting for variability in behavioural outcomes (Esposito et al., 2022). Therefore, further research is needed to clarify the synergistic effects of combined interventions simultaneously targeting cognitive performance and emotion regulation (indexed by psychophysiological measures) in novice mindfulness practitioners. The absence of a tDCS effect in this study may also be due to individual differences, including variations in brain anatomy, such as cortical thickness and volume (Razza et al., 2024), morphological and genetic features, and sex hormones or exogenous substance consumption (Vergallito et al., 2022) that should be explored as potential predictors of tDCS response in future studies. Furthermore, although F3/F4 montages mainly target the dlPFC, computational modeling suggests that peak current density may converge over the dorsomedial prefrontal cortex (dmPFC), a region closely linked to self-awareness and emotional regulation relevant to mindfulness (Choi & Lee, 2023).

The results showed no differences among groups based on self-report measures of state mindfulness. Previous research underscores that a mindfulness induction may not have an immediate effect on the self-perception of state mindfulness, particularly in novices (Lee & Orsillo, 2014; Leyland et al., 2019). Alternatively, an induction of VR-MW could be perceived as a mindfulness session for novice practitioners (Girardeau et al., 2020).

Nevertheless, a decrease in arousal was observed across all groups at the end of the session, with the combination of focused-mindful and tDCS showing a more pronounced decrease compared to the stand-alone VR-FM, tDCS, and control groups. Although we need caution due to the limited statistical power of the analysis, the significant difference observed between VR-FM + tDCS and VR-MW + tDCS sham suggests that the synergistic effects decrease emotional arousal. The relationship between mindfulness and skin conductance has yielded mixed results in the literature. Cosme and Wiens (2015) reported no significant differences in SCRs between meditators and novices in response to emotional stimuli, whereas Costa et al. (2020) found decreases in electrodermal activity following meditation in a nature-inspired VR environment.

This study has some limitations that merit our attention. First, participants were exclusively university students, limiting the generalisability of findings to other demographics. Second, we did not collect mindfulness-state at baseline, preventing us from determining whether the intervention successfully induced a state of mindfulness. Future studies should include pre- and post-intervention mindfulness assessments to measure changes attributable to the intervention, and incorporating brain imaging methods to determine whether the target brain regions were successfully stimulated, providing additional insights into its underlying mechanisms. Furthermore, Boucsein et al. (2012) highlight the need for baseline recording in skin conductance response for meaningful comparisons, a step our study overlooked. Finally, a notable limitation of our study is related to the simultaneous use of tDCS and a VR headset, which imposed restrictions by not allowing orbitofrontal electrode positioning.

We used MW as a control condition for the mindfulness intervention and tDCS sham as a control condition for the active tDCS condition. To ensure consistency across the study, we standardised the experimental setting for all participants. While such a factorial design is aimed to assess interactions and individual effects of independent interventions, it does not allow for isolating the effects of monotherapy, limiting our ability to assess the additive or combined effects of these interventions. Future studies could consider alternative designs to better evaluate the synergistic effects of these two therapeutic methods, including the addition of groups receiving only VR-FM or only tDCS. Furthermore, our study focused on novice mindfulness practitioners and was conducted in a VR environment to standardise participants’ engagement and ensure they focused on the same task. Research should also explore the effects of these interventions on expert mindfulness practitioners, as their advanced experience might reveal different outcomes and offer deeper insights into the mechanisms at play. To deepen the understanding of how these interventions work, future studies should integrate neuroimaging techniques to detect subtle or underlying brain changes that may not be evident through behavioural measures alone. In addition, incorporating participant profiling strategies, such as biomarker and genomic assessments, could help identify individual differences in responsiveness, paving the way for more personalised and effective applications of VR-FM and tDCS (Park et al., 2025; Pellegrini et al., 2021). Together, these improvements could build upon our findings and further advance the understanding of how these interventions enhance cognitive functions across diverse populations.

## Conclusion

Our randomised, double-blind, sham-controlled study found that a single session of combined VR-FM and anodal tDCS over the left dlPFC (anode at F3, cathode at F4) may not produce significant effects on attentional outcomes and inhibitory control in a non-clinical sample. These results highlight the complexity of cognitive interventions, namely in what concerns specific cognitive functions, their measures, and the interaction between cognitive and psychophysiological aspects.

We have used MW as a control condition for the mindfulness intervention and tDCS sham as a control condition for the active tDCS condition. To ensure consistency across the study, we standardised the experimental setting for all participants. While such a factorial design is aimed to assess interactions and individual effects of independent interventions, it does not allow for isolating the effects of monotherapy, limiting our ability to assess the additive or combined effects of these interventions. Future studies could consider alternative designs to better evaluate the synergistic effects of these two therapeutic methods, including the addition of groups receiving only VR-FM or only tDCS. Additionally, studies involving multiple sessions may help explore potential cumulative and long-term effects and allow for comparisons with single-session designs to better understand the efficacy of each intervention.

This study was supported in-kind by Sooma tDCS™, which provided two Sooma tDCS stimulators on loan free of charge.

## Funding

This work was supported by Bial Foundation [grant 323/2024].

AGA is supported by the Portuguese Foundation for Science and Technology (FCT) Grants 2020.02059.CEECIND (https://doi.org/10.54499/2020.02059.CEECIND/CP1609/CT0015). The Center for Research in Neuropsychology and Cognitive and Behavioral Intervention (CINEICC) of the Faculty of Psychology and Educational Sciences of the University of Coimbra is funded by national funds through FCT – Fundação para a Ciência e a Tecnologia, I.P., under the project/support UID/00,730. MS is supported by a doctoral research grant from FCT (project reference 2021.07006.BD; DOI identifier: https://doi.org/10.54499/2021.07006.BD).

## Declaration of competing interest

Perianen Ramasawmy reports a relationship with University Medical Center Göttingen Neurology Clinic that includes: employment.

Perianen Ramasawmy does not have any conflict of interest regarding the current work. P. Ramasawmy has received honorarium for teaching from NeuroCare (Germany) and is supported by the EU-Horizon 2020 (101,057,367; PAINLESS). If there are other authors, they declare that they have no known competing financial interests or personal relationships that could have appeared to influence the work reported in this paper.

Ana Ganho Avila reports financial support was provided by BIAL Foundation. Ana Ganho Avila reports equipment, drugs, or supplies was provided by Sooma tDCS TM. Ana Ganho Avila reports a relationship with Flow Neuroscience that includes: non-financial support. Member of the Advisory Board of the IJCHP; Secretary of the Board of the European Society for Brain Stimulation; Vice- President of the Neuromodulation Section of the Portuguese Society of Psychiatry and Mental Health If there are other authors, they declare that they have no known competing financial interests or personal relationships that could have appeared to influence the work reported in this paper.
